# Psychological Distress and Quality of Life in a Community-Based Sample of Adults with Atopic Dermatitis: A Cross-Sectional Exploratory Study

**DOI:** 10.3390/healthcare14030398

**Published:** 2026-02-05

**Authors:** Florence Mei Fung Wong, Pui Ka Fung, Hoi Yan Mak, Richard Yi Tsun Kao

**Affiliations:** 1 School of Nursing, Tung Wah College, Hong Kong SAR, China; 2 Department of Microbiology, The University of Hong Kong, Hong Kong SAR, China; ja713168@connect.hku.hk (P.K.F.); karenmhy@hku.hk (H.Y.M.); rytkao@hku.hk (R.Y.T.K.)

**Keywords:** atopic dermatitis, anxiety, depression, quality of life

## Abstract

**Highlights:**

**What are the main findings?**

- People with atopic dermatitis are experiencing physical and psychological distress.
- Strategies should be developed to tackle not only physical burden but also psychological distress.

**What are the implications of the main findings?**

- The strong negative associations between psychological distress and all quality-of-life domains highlight the need for integrated care.
- Our finding that inadequate sleep is significantly associated with lower environmental QoL suggests that addressing sleep disturbances could be a specific, modifiable target for improving well-being in this population.

**Abstract:**

**Background/Objectives**: Atopic dermatitis (AD) is a chronic inflammatory skin condition associated with psychological distress and reduced quality of life (QoL). The complex interrelationships among anxiety, depression, and multidimensional QoL in adults with AD remain insufficiently understood. This study aimed to examine these relationships and key factors linking psychological distress and QoL in this population. **Methods**: In this cross-sectional study, 47 adult participants with AD completed the Hospital Anxiety and Depression Scale (HADS) and the World Health Organization Quality of Life (WHOQOL-BREF). Bivariate and multivariate analyses were used to identify associations and correlates among anxiety, depression, and QoL domains. **Results**: Participants demonstrated elevated anxiety (mean score: 7.91 ± 3.27) and depression (mean score: 6.28 ± 3.62) scores, with moderate-to-poor QoL reported across all domains. Both anxiety and depression were significantly negatively correlated with all QoL dimensions (*p* < 0.05). Higher depression and self-reported stress were independently associated with poorer self-perceived QoL (*p* < 0.001). Self-reported inadequate sleep was associated with lower environmental QoL (*p* = 0.006), while more self-reported frequent AD flares correlated with reduced psychological QoL (*p* = 0.007). **Conclusions**: This study highlights the substantial psychological burden and impaired QoL experienced by adults with AD. Self-reported stress and inadequate sleep were commonly cited as modifiable factors associated with poorer outcomes, alongside elevated depression scores. An integrated care approach addressing both physical and psychological factors is recommended to improve overall outcomes in this population. Future research should prioritize longitudinal designs and AD-specific assessments to further elucidate causal pathways and inform targeted interventions.

## 1. Introduction

Atopic dermatitis (AD) is a chronic, relapsing inflammatory skin disorder that affects individuals across the lifespan, with a particularly high prevalence among children and young adults [1,2]. The condition is characterized by a range of physical symptoms, including xerosis, erythematous papules and plaques, lichenification, and severe pruritus [2,3]. These manifestations often lead to significant physical discomfort and functional impairment, which in turn adversely affect psychological and social well-being, ultimately diminishing overall quality of life (QoL) [4].

Evidence highlights that individuals with AD experience profound physical and psychological distress, an emotional state encompassing symptoms of both anxiety and depression [2,3]. Systematic reviews reports that adults with AD are at significantly elevated risk for comorbid anxiety and depression, with prevalence estimates ranging from 20% to 30%, substantially higher than those observed in the general population [4,5,6]. The burden of AD extends to sleep disturbances, social withdrawal, stigmatization, and reduced health-related QoL across physical, psychological, social, and environmental domains [7,8]. This relationship is bidirectional: psychological distress can exacerbate pruritus and inflammation through neuroimmune pathways, while poorly controlled symptoms perpetuate emotional and social dysfunction [7,8,9,10]. Moreover, AD can lead to significant functional impairments, including reduced work productivity, impaired concentration, and in severe cases, self-harm or suicidal ideation [9,10,11].

The management of AD remains clinically challenging, often requiring prolonged, multifaceted regimens that are burdensome in themselves [12]. Visible skin lesions frequently contribute to social stigmatization, diminished self-esteem, and avoidance of social and occupational activities [13,14,15]. During flares, patients may withdraw further, intensifying feelings of isolation [16,17]. This multifaceted burden underscores the need to better understand the interplay between psychological distress and multidimensional QoL in adults with AD.

Despite growing recognition of the interconnected physical and mental health impacts of AD, few studies have comprehensively examined the associated factors and pathways linking psychological distress to multidimensional QoL in community-based, non-clinical adult populations, particularly within Asian contexts. Young adults represent a critical group in whom AD may disrupt psychosocial development, yet targeted research in this age group remains limited. Therefore, this study aimed to address these gaps by examining these relationships in a community-based sample of predominantly young adults in Hong Kong. Our specifical objectives were to: (1) examine levels of anxiety, depression, and QoL in adults with AD, (2) explore associations between anxiety, depression, and QoL across physical, psychological, social, and environmental domains, (3) identify factors associated with anxiety, depression, and QoL outcomes.

Based on the existing literature, we hypothesize that:

**H1:**  *Adults with AD will report elevated levels of anxiety and depression compared to general population norms.*

**H2:**  *Higher levels of anxiety and depression will be significantly associated with poorer QoL across all domains (physical, psychological, social, and environmental).*

**H3:**  *Self-reported factors such as stress, inadequate sleep, and higher AD flare frequency will be associated with greater psychological distress and reduced QoL.*

## 2. Materials and Methods

### 2.1. Design

A cross-sectional study was conducted.

### 2.2. Subject Recruitment and Settings

Eligible participants were recruited from the community using convenience and snowball sampling methods to enhance accessibility.

#### 2.2.1. Selection Criteria

Participants were required to have a formal diagnosis of AD. Individuals with a pre-existing, formally diagnosed mental health condition (e.g., major depressive disorder, generalized anxiety disorder) were excluded. This criterion was implemented to minimize the confounding influence of significant, independent psychiatric comorbidity on the measurement of AD-associated psychological distress. The aim was to better isolate the psychological burden directly attributable to AD itself. We acknowledge that this exclusion may limit the generalizability of our findings to the broader AD population, which includes individuals with comorbid mental health diagnoses.

#### 2.2.2. Sample Size Calculation

The sample size calculation was performed based on a medium effect size (f^2^ = 0.39), derived from a previous study [16]. With a significance level of 0.05 and a desired power of 80%, a minimum of 46 participants was required.

### 2.3. Instruments

#### 2.3.1. Demographics

A form of demographic- and AD-related medical characteristics was used to collect data on age, gender, types of AD/eczema, duration of AD/eczema, family history of AD/eczema, and current treatments. The frequency of AD flares, defined as a self-perceived significant worsening of skin symptoms, including typically increased itch, redness, and/or rash, was also collected to help understand more about a change in daily management or the use of rescue medication.

To capture patient-identified factors associated with their condition, an open-ended question (“What factors do you believe are associated with your AD?”) was included. Narratives were collected and categorized into common domains such as self-reported stress, weather, allergies, environment, and inadequate sleep. These categorized items represent patient-perceived contributors and are not validated clinical measures of stress severity or sleep quality.

#### 2.3.2. The Chinese Version of the Hospital Anxiety and Depression Scale (HADS)

The Chinese version of the HADS, validated by Leung et al. [17], was used to assess anxiety and depression, which are commonly experienced psychological disturbances among individuals with chronic diseases. This 14-item instrument comprises two 7-item domains: anxiety (HADS-A) and depression (HADS-D). Each domain includes seven items rated on a 4-point Likert scale, ranging from 0 (never experiencing the symptom) to 3 (always experiencing the symptom), with total scores ranging from 0 to 21. Scores of ≥8 and ≥11 indicate borderline and abnormal levels of anxiety/depression, respectively. The instrument demonstrates good internal consistency, with Cronbach’s alpha values of 0.81 and 0.74 for the anxiety and depression subscales, respectively, indicating high reliability. This instrument presents good reliability with Cronbach’s alphas for anxiety, depression, and overall HADS at 0.765, 0.771, and 0.837, respectively.

#### 2.3.3. Hong Kong Chinese Version of the World Health Organization Quality of Life-Brief Version (WHOQOL-BREF)

The Hong Kong Chinese version of the WHOQOL-BREF questionnaire was employed to evaluate QoL in the study population. This self-reported instrument, validated by Leung et al. [18], comprises 24 items assessing four domains: physical health (7 items), psychological health (6 items), social relationships (3 items), and environment (8 items), as well as two individual items on overall QoL and general health. Scoring is based on a 5-point Likert scale, with total scores ranging from 0 to 100 following WHO’s scoring guidelines for the original WHOQOL-BREF. Higher scores indicate better QoL. The instrument demonstrates good reliability across all domains, with Cronbach’s alpha values ranging from 0.73 to 0.83. In this study, the instrument showed good reliability across all domains with Cronbach’s alphas ranging from 0.728 to 0.879.

### 2.4. Study Procedure

After ethics approval was obtained, eligible participants were recruited with an explanation of the study’s purpose and their role in the research. All participants provided written informed consent prior to data collection. Upon consent, participants completed a questionnaire packet comprising the demographic form, the Chinese HADS, and the Chinese WHOQOL-BREF.

### 2.5. Data Analysis

All statistical analyses were performed using IBM SPSS Statistics version 26. The normality of continuous variables was assessed using skewness statistics and normal Q-Q plots. Descriptive statistics were used to summarize the demographic and clinical characteristics of the participants, as well as the outcome variables of psychological well-being and QoL. Associations between psychosocial well-being, QoL, and demographic characteristics, were explored using Chi-square tests, bivariate analyses, and one-way ANOVA were employed. Given the sample size (*n* = 47), the multivariate regression analyses are considered exploratory. The number of associate factors in any given model was minimized to mitigate overfitting and ensure stable parameter estimates. Results from these analyses should be interpreted with caution as generating hypotheses for future research. All tests were two-sided, and statistical significance was defined as *p* < 0.05.

### 2.6. Ethical Considerations

Ethical approval was obtained from the Research Ethics Committee of the tertiary institution (Reference No: REC2024220). All participants received a full explanation of the study and provided written informed consent. Data were collected and stored anonymously to ensure confidentiality.

## 3. Results

### 3.1. Demographic Characteristics

Of 47 subjects, 59.6% was female. The mean age was 28.43 ± 9.30 years, with the majority (51.1%, *n* = 24) aged 18–25 years and 40.4% (*n* = 19) aged 26–35 years. Most participants (83.0%, *n* = 39) had been living with AD for over seven years, and nearly half (*n* = 21) reported longstanding disease. Regarding patient-perceived triggering factors, environmental influences by 66.0% (*n* = 31), with weather by 53.2% (*n* = 25), and self-reported stress 25.5% (*n* = 12) were identified.

A total of 14 participants provided treatment data: all used topical therapies, with 85.7% (*n* = 12) using steroids during flare periods. Systemic medications with topical treatment were used by 35.7%. A detailed summary is shown in Table 1.

### 3.2. Anxiety, Depression, and QoL

Among the 47 participants with AD, the mean anxiety scores were 7.91 (SD = 3.27) and the mean depression score was 6.28 (SD = 3.62), respectively. When compared to reported Hong Kong general population norms for the HADS [18], where the mean anxiety score was approximately 5.2 and depression 3.8, our AD sample displayed notably higher levels of psychological distress, supporting the observation of an elevated burden in this population.

Based on the WHOQOL-BREF, most participants reported moderate overall perceived QoL and general health. Domain-specific scores indicated predominantly moderate physical and psychological health, while social and environmental health were rated as moderate to high. Detailed information is shown in Table 2.

### 3.3. Associations Between Anxiety, Depression, and QoL Domains

Bivariate analyses revealed significant correlations between anxiety and all quality-of-life domains. Anxiety had the strongest significant association with perceived QoL (r=−0.401, p=0.005). Depression was negatively correlated with all QoL domains, notably with self-perceived QoL (r=−0.647, p<0.001).

Anxiety and depression were strongly positively correlated (*r* = 0.533, *p* < 0.001), indicating intertwined psychological distress. The interrelationships among QoL domains were also significant (*p* < 0.001), highlighting their interconnectedness.

### 3.4. Factors Associated with Anxiety, Depression, and QoL Domains

Multivariate regression analyses were conducted to identify factors associated with anxiety, depression, and various domains of QoL. Given the exploratory nature of these analyses and the modest sample size (*n* = 47), the results should be interpreted with caution due to the increased risk of model instability and overfitting. These multivariate analyses were explicitly exploratory and hypothesis-generating; they should not be interpreted as identifying definitive causal ‘key factors’. All regression coefficients are presented with 95% confidence intervals (CIs) in Table S2 to reflect the precision and uncertainty of the estimates.

#### 3.4.1. Anxiety

Higher levels of anxiety were significantly associated with higher depression (B=0.481, SE=0.114, 95% CI:0.251 to 0.710, p<0.001), with the model explaining a significant proportion of variance [F(1,45)=17.822, p<0.001].

#### 3.4.2. Depression

Depression was a significant factor of higher anxiety (B=0.270, SE=0.127, 95% CI:0.014 to 0.526, p=0.040). It was also significantly associated with poorer self-perceived QoL (B=−2.430, SE=0.550, CI:−3.540 to−1.321, p<0.001) and lower environmental QoL (B=−1.888, SE=0.776, CI:−3.540 to−0.323, p=0.019). The overall model was significant [F(3,43)=18.750, p<0.001].

#### 3.4.3. Self-Perceived QoL

Self-perceived QoL was adversely affected by depression (B=−0.098, SE=0.020, CI:−0.138 to−0.058, p<0.001), stress (*B* = −0.538, *SE* = 0.165, 95% *CI*: 0.205 to 0.871, *p* = 0.002), and allergy (B=−0.503, SE=0.248, CI:0.002 to 1.004, p=0.049). Conversely, better self-perceived health was positively associated with self-perceived QoL (*B* = 0.433, *SE* = 0.091, 95% *CI*: 0.250 to 0.615, *p* < 0.001). The model was significant (*F*(4,42) = 18.935, *p* < 0.001).

#### 3.4.4. Self-Perceived Health

Self-reported health was negatively associated with stress (*B* = −0.830, *SE* = 0.216, 95% *CI*: −1.267 to −0.394, *p* < 0.001) and allergy (*B* = −0.678, *SE* = 0.330, 95% *CI*: −1.344 to −0.012, *p* = 0.046). The overall model was significant (*F*(3,43) = 13.192, *p* < 0.001).

#### 3.4.5. Psychological QoL

Psychological QoL was positively associated with environmental QoL (*B* = 0.421, *SE* = 0.114, 95% *CI*: 0.196 to 0.650, *p* < 0.001) and negatively associated with the self-reported frequency of AD flares (*B* = −0.068, *SE* = 0.024, 95% *CI*: −0.117 to −0.019, *p* = 0.007). The model was significant (*F*(2,44) = 10.987, *p* < 0.001).

#### 3.4.6. Social QoL

Social QoL was positively associated with environmental QoL (*B* = 0.442, *SE* = 0.133, 95% *CI*: 0.174 to 0.710, *p* = 0.002) with significant model (*F*(1,45) = 11.014, *p* = 0.002).

#### 3.4.7. Environmental QoL

Environmental QoL was positively associated with physical QoL and social QoL but had significant negative association with inadequate sleep (*B* = −0.472, *SE* = 0.162, 95% *CI*: −0.799 to −0.146, *p* = 0.006). The overall model was significant (*F*(4,42) = 8.778, *p* < 0.001).

It is important to note that these multivariate models, conducted with a sample size of 47, were considered strictly exploratory and hypothesis-generating. The number of analyses performed relative to the sample size increased the risk of unstable estimates and spurious findings. The detailed regression coefficients, including 95% confidence intervals and significant values, are provided in Table S2. Results should be interpreted as hypothesis-generating rather than conclusive.

## 4. Discussion

This cross-sectional study examined psychological distress and QoL among adults with AD. The sample predominantly consisted of young adults (aged 18–35 years), a population of particular concern given the potential for AD to disrupt ongoing physical and psychosocial development [7]. Notably, 72.7% of participants had lived with AD for over a decade, and more than half reported experiencing more than 10 AD flares annually, with many exceeding 30 flares. This high burden of chronic and recurrent disease was reflected in participants’ self-rated health, which 63.8% described as moderate to poor. Commonly reported triggers for exacerbations included weather (53.2%), self-reported stress (25.5%), and other environmental factors, consistent with established psychosocial and climatic influences on AD activity [19,20].

The study population exhibited clinically relevant levels of anxiety (mean score = 7.91) and depression (mean score = 6.28). These scores may represent a “normalized high” baseline of psychological distress integrated into daily life, as most participants were not experiencing an active flare at the time of assessment. Pruritus, a core symptom, is strongly linked to mental health disturbances, particularly depression, and can act as a persistent stressor even during quiescent periods [20,21,22]. Depressive symptoms may, in turn, impair self-management, reduce treatment adherence, and worsen disease control, creating a bidirectional cycle linking skin and mental health [7,22,23].

QoL was reported as moderate to poor across all measured domains. Chronic physical symptoms like pruritus, erythema, and skin damage interfere with daily activities, sleep, and consistent treatment use [24]. Even in remission, residual skin discomfort and altered integrity necessitate ongoing management. The psychological domain was notably impaired, likely due to the impact of visible lesions on body image, self-esteem, and emotional well-being [24,25,26]. This psychological burden can undermine social relationships by reducing satisfaction with interpersonal connections and limiting access to support [25,26], while also affecting self-management, work performance, and productivity [27]. Environmental health encompassing safety, accessibility, and social infrastructure, was also rated poorly, suggesting broader contextual factors compound daily challenges.

Our findings indicate associations rather than causal relationships, consistent with the cross-sectional design of our study. Bivariate analyses revealed strong intercorrelations among all QoL domains, along with significant negative associations between anxiety/depression and each domain. Depression showed particularly robust negative correlations with overall self-perceived QoL and physical, psychological, social, and environmental health. These findings underscore the pervasive impact of mood disturbances on multidimensional well-being in AD. The interconnectedness of QoL domains suggests that deterioration in one area, often beginning with physical discomfort, can propagate to others, highlighting the need for holistic intervention [28,29].

Multivariate regression identified several modifiable factors associated with anxiety, depression, and QoL. Anxiety significantly associated with depression, and vice versa, supporting their close interplay in AD. Depression also associated with poorer self-perceived and environmental QoL. Self-perceived QoL was adversely influenced by depression, self-reported stress, and allergies, but positively associated with self-rated health [21,30]. Environmental QoL was positively linked to physical, psychological, and social QoL but negatively associated with inadequate sleep. Notably, the strong correlation between sleep disturbances and lower environmental QoL suggests that sleep hygiene may be a specific target for intervention to improve overall well-being. Furthermore, psychological QoL was negatively affected by self-reported AD flare frequency, emphasizing the cyclical relationship between disease activity and mental well-being [7].

Since physical symptoms, particularly pruritus, are closely associated with distress and reduced QoL, optimizing symptom control should be a primary therapeutic goal. Addressing sleep disturbances and stress is also crucial, given their association with lower environmental QoL and potential to exacerbate AD severity and mental health symptoms [18,19]. Our data specifically highlight that self-reported inadequate sleep correlates with lower environmental QoL and higher disease frequency, underscoring sleep hygiene as a modifiable target within this cycle of symptoms and distress. Environmental modifications, alongside psychosocial support, could help disrupt the itch–scratch–stress–flare cycle [7]. Recognizing the interrelated roles of self-perceived health, self-reported stress, and comorbidities such as allergy is essential. Integrated care models combining dermatological treatment with psychological support, sleep hygiene education, and environmental counseling may be particularly beneficial. A multidimensional, patient-centered approach addressing physical, emotional, social, and environmental needs is recommended to improve overall well-being in this population.

This study has several important limitations. First, its cross-sectional design precludes causal inference, limiting our ability to determine temporal relationships between variables. Second, the modest sample size (*n* = 47), while sufficient for primary bivariate analyses, restricts the power and stability of the multivariate regression models. The relatively small number of participants limits the number of models we can reliably test without risking overfitting, and the wide 95% confidence intervals around many estimates reflect limited precision. Consequently, these results should be considered exploratory and hypothesis-generating rather than definitive.

Methodological constraints further qualify the findings. We relied on self-reported frequency of AD flares and perceived triggers without objective clinical severity measures (e.g., SCORAD, EASI, POEM). Therefore, it remains unclear whether the observed associations are mediated by objective disease severity or are influenced by subjective illness perceptions. Consequently, the reported links between psychological distress, self-reported factors, and QoL may be confounded by unmeasured disease severity. The use of generic instruments (HADS, WHOQOL-BREF), while validated, may not capture AD-specific nuances. Regarding treatment data, we acknowledge that the initial analysis did not thoroughly examine the impact of systemic versus topical treatments on QoL. Due to limited data (available for only 14 participants) and the small sample size within treatment categories, further analyses controlling for treatment type may not produce reliable insights. Therefore, future research is essential to explore the role of treatment modalities more comprehensively.

Finally, the use of convenience and snowball sampling introduces selection bias. Participants may share characteristics (e.g., higher healthcare engagement, social connectivity) not representative of the general AD community, further constraining the external validity and generalizability of our results. Future research should employ longitudinal designs, incorporate validated AD-specific and severity measures, include participants across the spectrum of mental health conditions, and utilize population-based sampling to verify and extend these findings.

## 5. Conclusions

This study has demonstrated a substantial burden of psychological distress and impaired QoL among individuals with AD, while identifying key associations and contributing factors. The findings indicate that adults with AD commonly report elevated levels of anxiety and depression, alongside moderate to poor QoL across physical, psychological, social, and environmental domains. The chronic and relapsing course of AD results in persistent physical symptoms, compromised skin integrity, and altered appearance, which are the factors that collectively contribute to diminished psychological well-being, social engagement, and satisfaction with environmental conditions.

Our exploratory analyses further identified specific, potentially modifiable factors, such as self-reported inadequate sleep and higher self-reported AD flare frequency, that were associated with poorer outcomes. These findings provide a focused foundation for hypothesis generation and future intervention development. For example, integrating sleep hygiene and stress management support alongside dermatological treatment could be a promising approach to address key areas of patient concern. These preliminary insights may offer valuable guidance for healthcare professionals in designing more integrated, holistic care models that address both the dermatological and psychosocial dimensions of AD. However, due to methodological limitations, including modest sample size, a cross-sectional design, and a community-recruited sample using convenience and snowball sampling from a predominantly young, highly educated cohort, these findings should be interpreted as preliminary evidence specific to this group and require confirmation in larger, more representative cohorts using longitudinal designs.

Future research should prioritize longitudinal designs and incorporate AD-specific assessment tools to better elucidate the dynamic relationship between disease activity and well-being over time. Confirmatory studies are needed to verify the stability and generalizability of the associations identified here. Ultimately, advancing more effective medical treatments to reduce physical symptom burden remains essential for breaking the cycle of symptom exacerbation and psychological distress, ultimately improving overall QoL in this population.

## Figures and Tables

**Table 1:** Demographic characteristics and AD-perceiving factors.

	Frequency	Percentage
Gender		
Male	19	40.4
Female	28	59.6
Age ranges (years old)		
18–25	24	51.1
26–35	19	40.4
36–45	1	2.1
>45	3	6.4
Educational level		
Primary or lower	0	0
Secondary	2	4.3
Tertiary or higher	45	95.7
Years obtained eczema		
≤5 years	6	12.6
6–≤10 years	7	14.7
>10 years	34	72.7
Eczema flares (per year)		
<1 to 5 times	19	40.4
6 to 10 times	1	2.1
11 to 20 times	4	8.5
21 to 30 times	2	4.3
>30 times	21	44.7
Related factors *		
Stress	12	25.5
Weather	25	53.2
Allergy	4	8.5
Environment	31	66.0
Inadequate sleeping	7	14.9
Current Treatment Types (*n* = 14)		
Topical	2	14.3
Steroids	12	85.7
System and Tropical	7	0.5

* Percentages for perceived related factors do not sum to 100%, as participants could select multiple options.

**Table 2:** Quality of life based on WHOQOL-BREF (*n* = 47).

WHOQOL-BREF	Low (1)	Moderate (2)	High (3)
Perceived QoL	7 (14.9)	18 (38.3)	22 (46.8)
Perceived health	17 (36.2)	15 (31.9)	15 (31.9)
Physical health	3 (6.4)	34 (72.3)	10 (21.3)
Psychological health	5 (10.6)	37 (78.7)	5 (10.6)
Social health	0	26 (55.3)	21 (44.7)
Environmental health	0	25 (53.2)	22 (46.8)

1 = low, 2 = moderate, 3 = high.

## Data Availability

The data presented in this study are available on request from the corresponding author. The data are not publicly available due to keep the confidentiality.

## Supplementary Materials

The following supporting information can be downloaded at: https://www.mdpi.com/article/10.3390/healthcare14030398/s1, Table S1: Associations among HADS and QoL domains, Table S2: Factors associated with anxiety, depression, and QoL domains.

## Abbreviations

The following abbreviations are used in this manuscript:

AD	Atopic Dermatitis
HADS	Hospital Anxiety and Depression Scale
QoL	Quality of Life
WHOQOL-BREF	World Health Organization Quality of Life-Brief Version
