# Supplementary material for: Psychological Distress and Quality of Life in a Community-Based Sample of Adults with Atopic Dermatitis: A Cross-Sectional Exploratory Study
Source: Healthcare (Basel). 2026 Feb 5;14(3):398. doi: 10.3390/healthcare14030398 (PMC12897151; doi:10.3390/healthcare14030398)
Supplement: Supplementary file 1 [file healthcare-14-00398-s001.zip › healthcare-4088134-supplementary.pdf]

Table S1. Associations among HADS and QoL domains

	Anxiety		Depression		Perceived QOL		Perceived Health		Physical Health		Psychological Health		Social Health		Environmental Health	
	γ	p	γ	p	γ	p	γ	p	γ	p	γ	p	γ	p	γ	p
<u>HADS</u>																
Anxiety	1		0.533	<0.001***	-0.401	0.005*	-0.153	0.304	-0.305	0.037*	-0.314	0.032*	-0.373	0.010*	-0.358	0.014*
Depression	0.533	<0.001***	1		-0.647	<0.001***	-0.306	0.037*	-0.364	0.012*	-0.412	0.004**	-0.380	0.008*	-0.465	<0.001***
<u>WHOOL-Brief</u>																
Perceived QoL	-0.401	0.005*	-0.647	<0.001***	1		0.527	<0.001***	0.599	<0.001***	0.665	<0.001***	0.332	0.023**	0.381	0.008*
Perceived health	-0.153	0.304	-0.306	0.037*	0.322	0.027**	1		0.528	<0.001***	0.389	0.007*	0.138	0.355	0.437	0.002**
Physical domain	-0.305	0.037*	-0.364	0.012*	0.339	0.020**	0.322	0.027**	1		0.749	<0.001***	0.464	0.001**	0.479	<0.001***
Psychological domain	-0.314	0.032*	-0.412	0.004**	0.665	<0.001***	0.389	0.007*	0.749	<0.001***	1		0.696	<0.001***	0.598	<0.001***
Social domain	-0..373	0.010*	-0.380	0.008*	0.332	0.023**	0.138	0.355	0.464	0.001**	0.696	<0.001***	1		0.419	0.003**
Environment domain	-0.358	0.014*	-0.465	<0.001***	0.381	0.008*	0.437	0.002*	0.479	<0.001***	0.598	<0.001***	0.419	0.003**	1	

γ: Pearson correlation; *p<0.05, **p<0.005, ***p<0.001

Table S2. Factors associated with anxiety, depression, and QoL domains

	Anxiety				Depression			
	B	SE	95% CI	p	B	SE	95% CI	p
Anxiety					0.270	0.127	0.014, 0.526	0.040**
Depression	0.481	0.114	0.251, 0.710	<0.001***				
Self-perceived QoL					-2.430	0.550	-3.540, -1.321	<0.001***
Environmental QoL					-1.888	0.776	-3.454, -0.323	0.019*
	Self-perceived QoL				Self-perceived health			
Depression	-0.098	0.020	-0.138, -0.058	<0.001***				
Self-perceived QoL					0.706	0.129	0.445, 0.966	<0.001***
Self-perceived health	0.433	0.091	0.250, 0.615	<0.001***				
Allergy	-0.503	0.248	0.002, 1.004	0.049*	-0.678	0.330	-1.344, -0.012	0.046*
Stress	-0.538	0.165	0.205, 0.871	0.002**	-0.830	0.216	-1.267, -0.394	<0.001***
	Physical QoL				Psychological QoL			
Environmental QoL	0.404	0.138	0.125, 0.682	0.005**	0.421	0.114	0.191, 0.650	<0.001***
Frequency of AD episodes					-0.068	0.024	-0.117, -0.019	0.007*
	Social QoL				Environmental QoL			
Physical QoL					0.267	0.123	0.019, 0.515	0.035*
Social QoL					0.324	0.125	0.072, 0.575	0.013*
Environmental QoL	0.442	0.133	0.174, 0.710	0.002**				
Inadequate sleep					-0.472	0.162	-0.799, -0.146	0.006**

*p<0.05, **p<0.005, ***p<0.001
